# Azelaic Acid in Dermatology: A Review of Its Mechanism of Action

**DOI:** 10.7759/cureus.94491

**Published:** 2025-10-13

**Authors:** Camilo Mariano-Rodriguez, Pamela Nava-Martinez, Valeria Lyzzete Diaz-Molina

**Affiliations:** 1 Dermatology, General Hospital of México “Dr. Eduardo Liceaga”, Mexico City, MEX; 2 Family Medicine, National Autonomous University of Mexico, Mexico City, MEX

**Keywords:** azelaic acid, clinical use, dermatology, mechanism of action, pharmacology

## Abstract

Azelaic acid (AzA) is a naturally occurring dicarboxylic acid initially studied for its role in skin hypopigmentation and later developed for treating hyperpigmentary disorders due to its tyrosinase inhibition. Over time, AzA has demonstrated therapeutic potential in various dermatological conditions due to its multifaceted mechanisms of action, which remain not entirely understood.

This incomplete understanding represents a significant limitation, as elucidating the molecular and cellular pathways involved may not only optimize its current clinical applications but also facilitate the identification of novel therapeutic indications. A comprehensive review of its mechanisms is therefore essential to support evidence-based use and encourage future research into its broader dermatological potential.

## Introduction and background

Azelaic acid (AzA) has become increasingly relevant in modern dermatology due to its multifaceted therapeutic applications, including acne vulgaris, rosacea, melasma, and other inflammatory or pigmentary skin disorders. Its broad spectrum of clinical efficacy and favorable safety profile have led to its incorporation into numerous treatment algorithms and guidelines.

Historically, AzA emerged from studies on skin surface lipids and the pathogenesis of hypochromia in pityriasis versicolor. It was later found that *Malassezia *species oxidize unsaturated fatty acids to C8-C12 dicarboxylic acids, which act as competitive inhibitors of tyrosinase in vitro. This discovery prompted the development of AzA as a topical agent for hyperpigmentary disorders [1]. Additionally, AzA is naturally found in grains such as wheat and rye and has been detected in the urine of healthy individuals as a product of fatty acid oxidation.

Despite its widespread clinical use, the precise mechanisms of action of AzA remain incompletely understood. Although several biological pathways have been described, they are often studied in isolation and without clear mechanistic integration. Moreover, recent data on novel formulations, delivery systems, and extended indications remain limited or scattered, highlighting a need for updated synthesis.

This review aims to bridge that gap by consolidating available evidence on AzA’s pharmacological actions and their relevance to specific dermatological conditions. By doing so, it seeks to provide a more comprehensive understanding of the compound’s therapeutic potential and to identify areas for future research and clinical innovation.

Methods

A comprehensive narrative literature review was conducted to explore the pharmacological properties and mechanisms of action of AzA. The search included peer-reviewed articles, reviews, and foundational texts across internationally recognized academic databases. No publication date filters were applied to ensure the inclusion of both early foundational studies and more recent evidence. Although recent studies were considered, it is important to note that detailed pharmacological information on azelaic acid remains scarce in the current literature.

## Review

Chemistry

**Figure 1:**
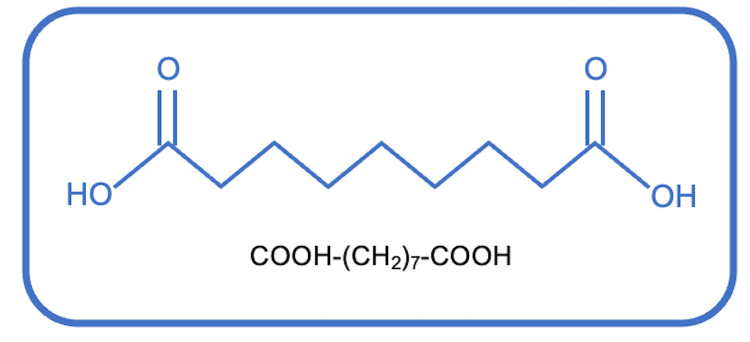
1,7-heptanedicarboxylic acid

Azelaic acid (1,7-heptanedicarboxylic acid) is a naturally occurring saturated nine-carbon dicarboxylic acid (COOH-(CH2)7-COOH) with a molecular weight of 188.22 g/mol [2] (Figure 1). Solubility in water varies according to temperature, but AzA is freely soluble in boiling water and alcohol. The chemical form of AzA currently used in dermatological products is obtained from *Malassezia furfur* [1] because of the fungus's lipoxygenases that can oxidize unsaturated fatty acids [3-5].

Pharmacokinetic profile

While AzA is available in various pharmaceutical forms, gel and cream remain the most commonly used formulations in clinical practice.

Percutaneous *absorption* after topical application of the 15% gel formulation has not been determined; however, for the 20% cream formulation, it amounts to 3.6% of the dermally applied dose [4,5]. *Systemic load* following percutaneous absorption for 15% AzA gel is 5.8% and 16.3% for 20% AzA cream [6-8]. Notably, despite lower systemic load, the gel formulation delivers a significantly higher fraction of the active ingredient into the viable skin layers compared to the cream (25.3% vs. 3.4%), highlighting its superior intradermal penetration and potential clinical advantage [6-8]. It is relevant to highlight that the gel formulation incorporates micronized azelaic acid, a modification that facilitates enhanced percutaneous absorption and improves transfollicular permeation kinetics [9]. In addition, liposomal gel formulations of AzA have been developed, offering further advantages as higher drug concentration in the stratum corneum compared to standard non-liposomal products as well as improved bioavailability [10].

The way AzA is transported across the cell membrane is still unknown but is thought to be through a transport carrier system or by simple diffusion. The existence of carnitine-mediated transport in the inner mitochondrial membrane has been proposed [11].

About 60% of the AzA absorbed systemically from the topical dose is *eliminated* mainly unchanged through renal excretion in the urine [4] in a period of 72 h. The remaining part of percutaneous absorbed AzA is broken down by* *β-oxidation into pimelic (heptanedioic) acid and glutaric (pentanedioic) acid (the major urinary metabolites) to acetyl CoA (which may enter *c*holesterol biosynthesis or Krebs cycle, being completely oxidized to CO_2_ and H_2_O) and malonyl CoA (precursor in fatty acid biosynthesis) [1,10,12].

It has been reported that in patients with ketosis and those with a congenital or acquired inability to β-oxidize monocarboxylic acids (dicarboxylic aciduria), AzA excretion can be altered [3].

AzA is part of the diet and, as said before, is formed endogenously; hence, plasma concentrations and daily urinary excretion are dependent on dietary intake. Nonetheless, 15% AzA gel does not increase plasma AzA concentration beyond the level derived from nutrition and endogenous metabolism [8]; thus, its toxicity risk is low. It is also important to emphasize that the pharmacokinetics of AzA is shaped by both the formulation and the barrier function of the skin.

The most frequent* adverse effect* is a mild transient erythema and cutaneous irritation characterized by scaling, pruritus, and a mild burning sensation [4]. Topical administration of 15% gel or 20% cream is well tolerated in humans, does not induce allergic sensitization or photodynamic skin reactions, and is devoid of overt adverse systemic effects [13]. However, AzA has been associated with rare occurrences of asthma, vitiligo depigmentation, small depigmented spots, hypertrichosis, and exacerbation of recurrent herpes labialis [5,6].

Although specific clinical data on the use of AzA during *pregnancy* and *lactation* are limited, no adverse effects have been reported to date. However, this drug has been classified under Pregnancy Category B [8,14].

Pharmacodynamic profile

The exact mechanisms of action of AzA in the treatment of dermatologic disorders are unclear, but several properties have been described that contribute to its efficacy observed in pilosebaceous disorders and pigmentation disorders [9,15].

Anti-inflammatory Action

AzA is a product of lipid oxidation, and because of that, it can bind to proliferator-activated receptors (PPARs). PPARs are ligand-dependent transcription factors that regulate target gene expression by binding to specific peroxisome proliferator-response elements. PPARγ is one of the three isoforms of PPARs identified in keratinocytes that inhibit cell proliferation and reduce the inflammatory responses. PPAR-γ represses NF-κB activation by physically interacting with p65 and p50 subunits, avoiding its translocation to the nucleus and thus inhibiting transcription of genes for proinflammatory cytokines such as IL-1β, IL-6, or TNFα [16,17]. In the same way, UVB radiation induces phosphorylation of p38 that modulates NF-κB activation, triggered by an elevation of reactive oxygen species (ROS) [16].

**Figure 2:**
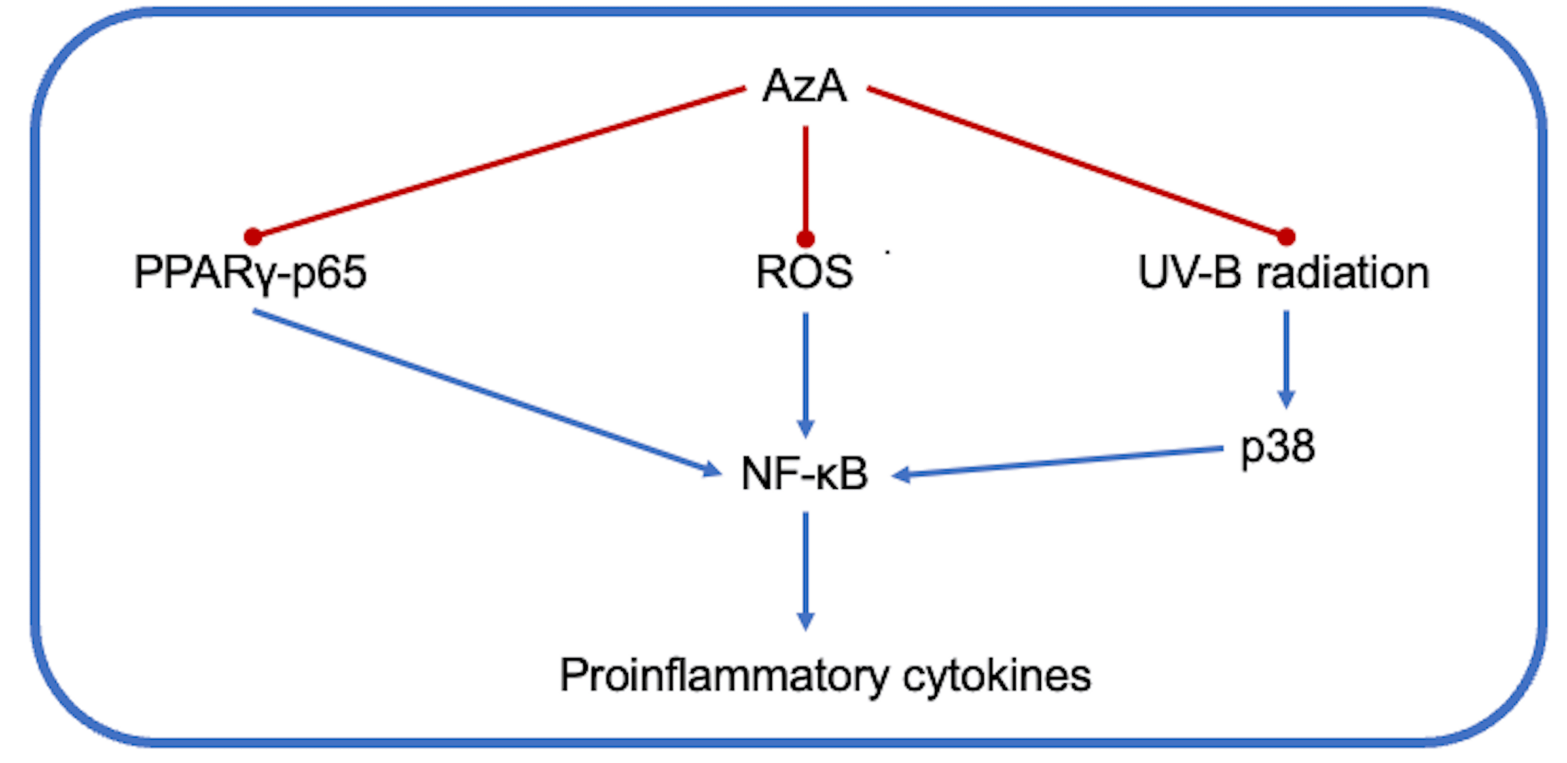
AzA inhibits cytokines biosynthesis based on the interference with NF-kB axis AzA: Azelaic acid; PPAR: Proliferator-activated receptor; ROS: Reactive oxygen species; NF-kB: Nuclear factor kappa B.

Mastrofrancesco et al. showed that AzA induces the expression of PPAR-γ (mRNA and transcriptional activity), significantly preventing the translocation of p65 subunit to the nucleus. Additionally, they demonstrated that UVB-induced IL-1β, IL-6, and TNF-α expression in human keratinocytes was significantly inhibited by AzA, which was explained by the inhibition of phosphorylation of p38. Furthermore, AzA showed moderate inhibitory effects on ROS formation in normal keratinocytes exposed to UVB irradiation, probably because of AzA's scavenging properties, thus preventing NF-κB activation (Figure 2) [16,17].

AzA directly inhibits kallikrein 5 (KLK5) and cathelicidin antimicrobial peptide gene (CAMPG) by decreasing their mRNA. CAMPG encodes a LL-37 peptide that has not only an antimicrobial effect but also a proinflammatory and angiogenic effect on the skin. KLK5 is a stratum corneum enzyme responsible for posttranslational processing of CAMPG. So, when AzA inhibits KLK5 and CAMPG and decreases LL-37 concentration, thereby reducing its proinflammatory and angiogenic effects on the skin, the antimicrobial effect is not really affected because of the antibacterial properties of AzA [18-20].

Antibacterial Action

AzA's effects on the physiology of cutaneous bacteria are dependent on the concentration of AzA, with higher concentrations being bactericidal; on the pH of the environment, with low pH enhancing the antimicrobial activity of azelaic acid; and on the nutrient status of the environment, with more nutrients offering protection to the bacteria, except at pH values below 6 [21].

AzA is actively and unselectively transported into the bacteria’s cytoplasm by ion transporters, causing a reduction of intracellular pH. Reducing pH from 6 to 4 results in a 14-fold increase in AzA uptake, so probably AzA transportation to bacteria’s cytoplasm could be pH-dependent [18].

Likewise, reduction of intracellular pH affects the maintenance of a pH gradient across the cell membrane and provokes loss of energy generation by bacterial metabolism such as respiratory chain (where reduced NADH dehydrogenase, succinic acid dehydrogenase, and reduced ubiquinone cytochrome-c oxidoreductase are inhibited) [18,21-24] and anaerobic glycolysis (which inhibits hexokinase) [6,25]. This low-energy environment decreases protein synthesis and RNA and DNA synthesis [26,18].

AzA also acts against several bacteria, including *Staphylococcus aureus*, *Staphylococcus capitis*, *Staphylococcus hominis*, *Escherichia coli*, *Corynebacterium diphtheriae*, *Proteus mirabilis*, *Pseudomonas aeruginosa*, *P. granulosum,* and* P. avidum*, though the mechanism is unclear [1,18].

Brasch and Christophers demonstrated the antimycotic activity of AzA and showed that growth of *Scopulariopsis brevicaulis*, *Epidermophyton floccosum*, *Trichophyton rubrum*, *Trichophyton mentagrophytes,* and other dermatophytes is completely inhibited by 0.56% AzA or higher concentrations. Also, *Candida albicans* and *Candida ​​​​​​glabrata* growth is inhibited by 4% AzA or higher concentrations, though *C. glabrata* appears to be more repressed by AzA than *C. albicans* [27].

**Figure 3:**
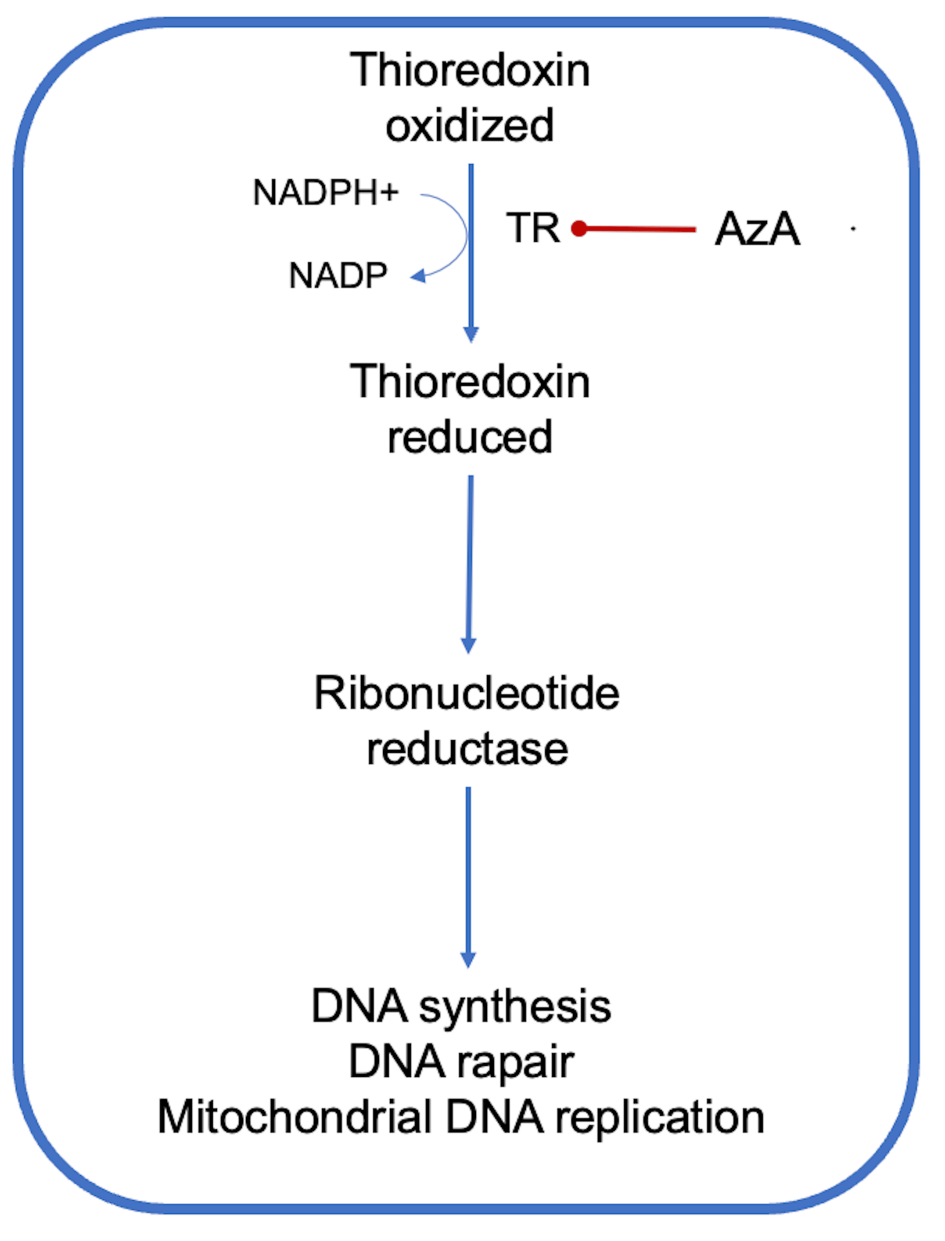
AzA competitively inhibits thioredoxin reductase, resulting in reduced ribonucleotide reductase activity, which in turn leads to decreased DNA synthesis and repair, as well as reduced mitochondrial DNA replication in multiple microorganisms, thereby exhibiting antibacterial properties AzA: Azelaic acid; NADPH: Nicotinamide adenine dinucleotide phosphate.

AzA has been shown to be an effective competitive inhibitor of thioredoxin reductase (TR) in *E. coli* and many microorganisms. This inhibition affects the TR/thioredoxin electron transfer system, the principal electron donor to the ribonucleotide reductases, thus preventing the biosynthesis of deoxyribonucleotides for DNA synthesis (Figure 3) [28,29]. In vitro studies reported that AzA leads to dose-related inhibition of *E. coli* DNA polymerase activity [14,30-32].

These modes of action do not cause any resistance in bacteria as antibiotics do, supporting the idea that AzA is a better option than topical antibiotics, making it as effective as topical antiseptics.

*Anti-melanogenesis* *Action*

**Figure 4:**
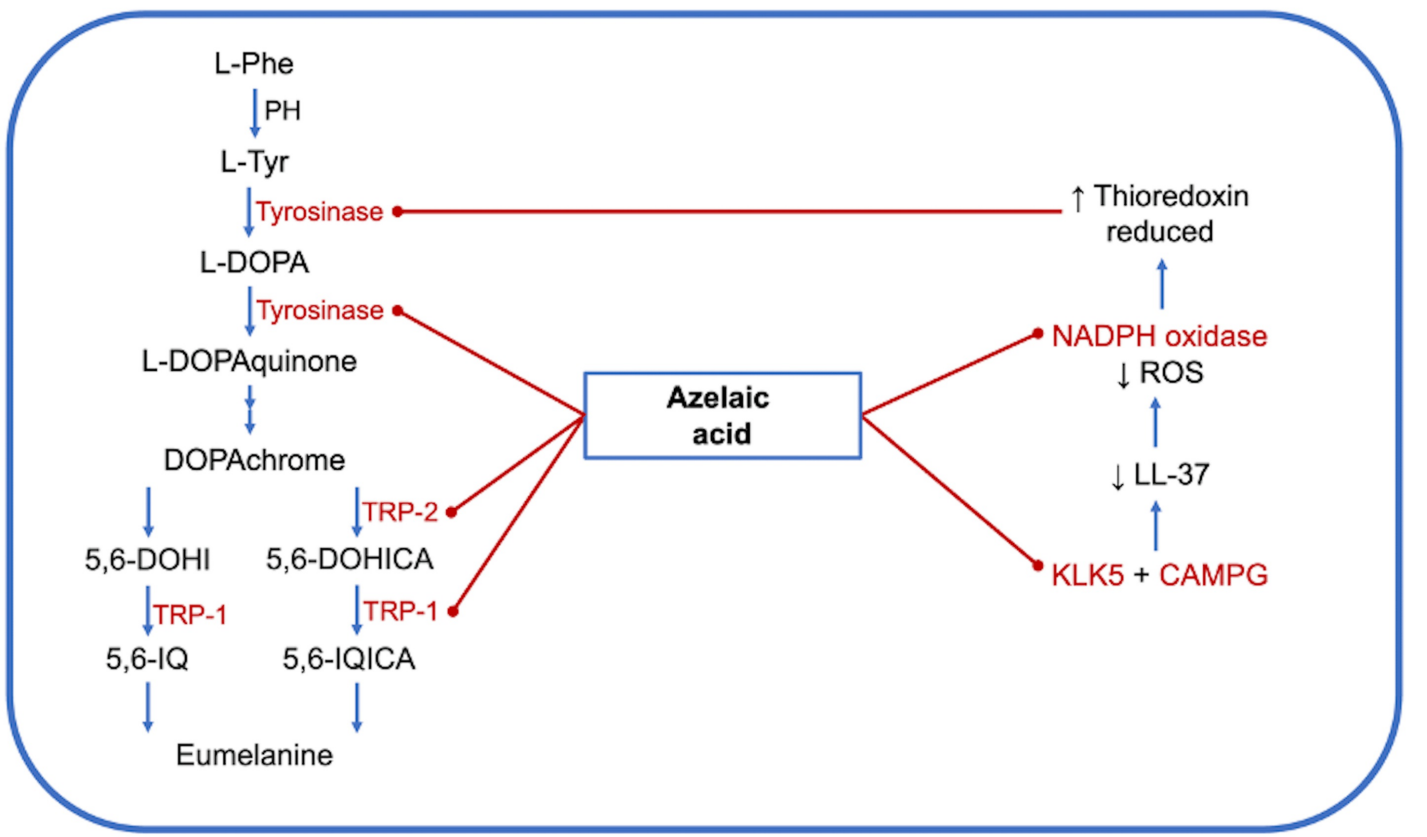
Anti-melanogenetic properties AzA acts as a competitive inhibitor of tyrosinase and competes with L-tyrosine for the α-carboxylate binding site on the enzyme's active site. It also inhibits NADPH oxidase in neutrophils, kallikrein 5 (KLK5), and the cathelicidin antimicrobial peptide gene (CAMPG), thereby reducing the generation of reactive oxygen species (ROS) and lowering the concentration of LL-37. These actions increase the intracellular concentration of reduced thioredoxin, a potent inhibitor of tyrosinase. Additionally, AzA decreases the expression of tyrosinase-related protein-1 (TRP-1) and tyrosinase-related protein-2 (TRP-2). NADPH: Nicotinamide adenine dinucleotide phosphate.

In vitro studies on melanoma cell lines showed that AzA interferes with DNA synthesis and mitochondrial enzymes and decreases tyrosinase activity by competitive inhibition [9,33,34], an effect that can be enhanced by the addition of zinc [18,35]. As mentioned above, AzA inhibits the TR/thioredoxin electron transfer system. This reductase has two functions in the epidermis surface: it reduces free radicals and regulates melanin biosynthesis. Melanogenesis is regulated by a feedback mechanism that involves nicotinamide adenine dinucleotide phosphate (NADPH), TR, thioredoxin, and tyrosinase [29]. Reduced NADPH produced in the cytosol from glycolysis transfers electrons to membrane-associated TR. Reduced TR can transfer electrons either to UV-generated free radicals outside the cell or to oxidized thioredoxin in the cytosol. When AzA scavenges free radicals (will be explained later in more detail), electrons flow in the direction of oxidized thioredoxin increasing the intracellular concentration of reduced thioredoxin, which is a potent inhibitor of tyrosinase, inhibiting melanin biosynthesis [28,29]. Another mechanism proposed for tyrosinase inhibition is that a single AzA carboxylate group competes with L-tyrosine for the α-carboxylate binding site on the tyrosinase active site (Figure 4) [36].

In cell culture, AzA has been shown to selectively penetrate tumoral cells [37] and to have a time- and dose-dependent antiproliferative and cytotoxic effect on human cutaneous melanoma [34], lymphoma, and leukemia-derived cell lines. On tumoral cells, AzA inhibits DNA synthesis and damages mitochondria; it also affects the karyotype of melanoma cells exposed to subtoxic doses in long-term culture [6].

Antikeratinizing Action

AzA also has an antikeratinizing effect [9,18] by arresting keratinocytes in the G0 phase [16]. With electron microscopy and immunocytochemistry, it has been observed that AzA particularly affects terminal phases of keratinization by reducing the size and number of keratohyalin granules and tonofilament bundles [9,26].

**Figure 5:**
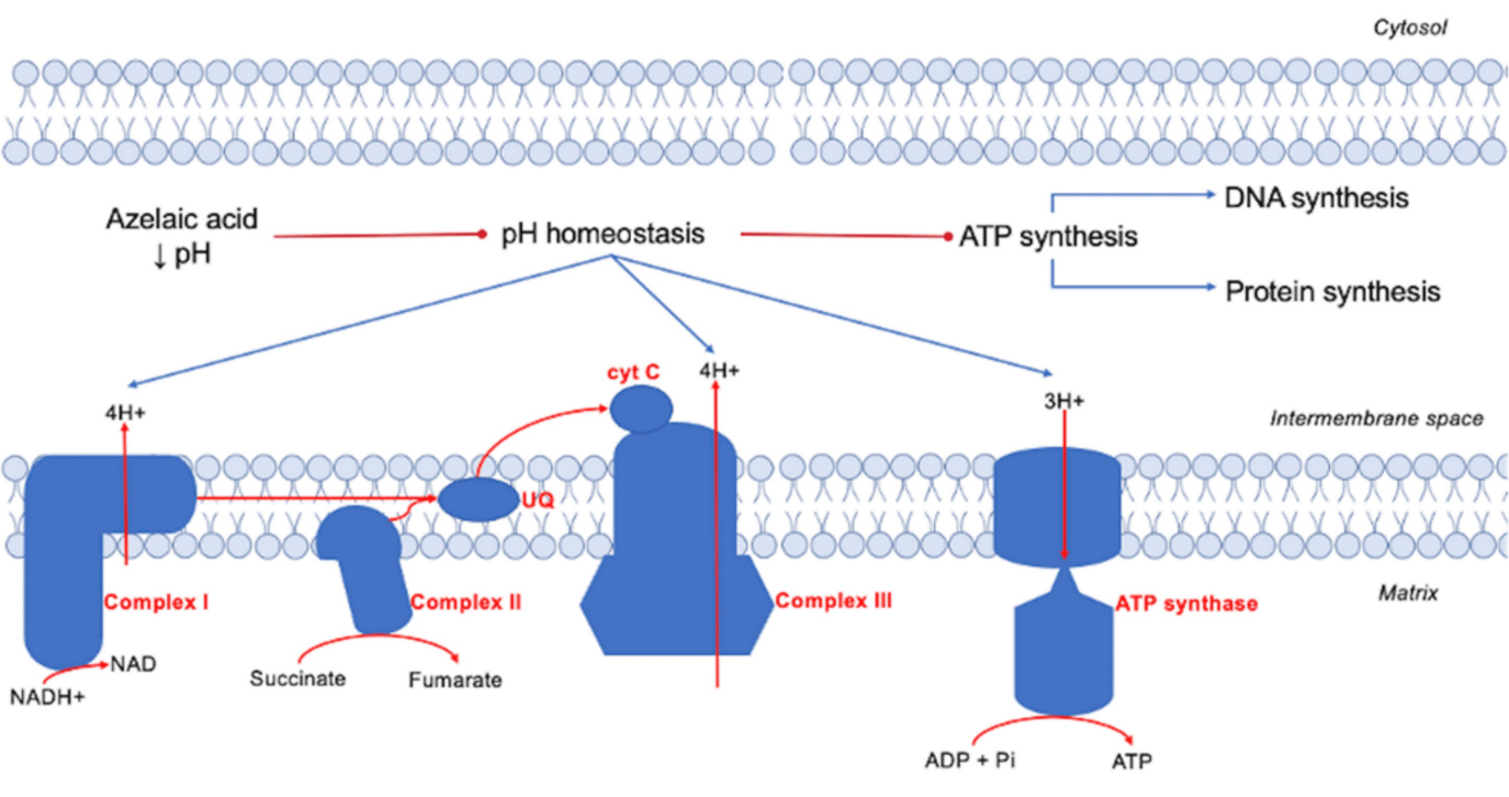
AzA competitively inhibits the respiratory chain and interferes with oxidative phosphorylation, affecting DNA and protein synthesis and causing swelling of the mitochondria and enlargement of the rough endoplasmic reticulum in the keratinocytes

AzA has been proposed to exert an antiproliferative effect on keratinocytes by decreasing keratinocyte DNA synthesis in a dose- and time-dependent manner [18]. Antimitochondrial effects of AzA are explained by a competitive inhibition of the following enzymes: NADH dehydrogenase, succinic dehydrogenase, and cytochrome-c oxido-reductase [38], causing swelling of the mitochondria and enlargement of the rough endoplasmic reticulum in the keratinocytes [9]. Consequently, ATP synthesis decreases, which affects the signaling cascade of ATP-dependent kinases such as fibroblast growth factor receptor-2b (FGFR2b) and decreases DNA synthesis (Figure 5).

FGFR2b is localized mainly in the suprabasal spinous layer of the epidermis and sebocytes; it has three main downstream signaling cascades that are affected by AzA: the mitogen-activated protein kinase (MAPK) pathway (resulting in decreased cell proliferation and matrix metalloproteinase expression), the phosphatidylinositide 3-kinase/Akt (PI3K/Akt) and sonic hedgehog/melanocortin-5 receptor pathway (decreasing lipogenesis and terminal sebocyte differentiation), and the phospholipase Cg/protein kinase-C pathway (decreasing IL-1a and inflammatory reactions) [39], those actions could explain AzA effect on acne and papulopustular rosacea.

In the stratum granulosum, AzA reduces filaggrin expression [18,40], increases the number of lamellar bodies, reducing adhesion of the horny cells [9,18], and enhances local antimicrobial immunity. As a result, AzA has an inhibitory effect on the generation of comedones and improves homeostasis of the epidermis.

Free-Radical Scavenging Activities

AzA can scavenge ROS released by neutrophils, and it may have a physiological role as a natural antioxidant [12,18]. Neutrophil production of ROS is mediated by NADPH oxidase activity (located on the surface membrane of neutrophils), which is effectively inhibited by lower concentrations of AzA [23].

Particularly, scavenging of ·OH gains importance in the peroxidation of arachidonic acid, which reduces inflammation [41,42]. In the same way, AzA in vitro can inhibit the hydroxylation of L-tyrosine to L-DOPA that requires ·OH produced by the Fenton reaction [1].

LL-37 stimulates the generation of ROS through NADPH oxidase activation and intracellular calcium mobilization, so inhibition of LL-37 by AzA reduces ROS generation [23].

5-Alpha-Reductase Inhibition

Stamatiadis et al. reported that AzA can competitively inhibit 5-alpha-reductase, which converts testosterone to dihydrotestosterone in both human skin and hair follicles. It has been proposed that AzA could competitively occupy the NADPH-binding site of 5a-reductase, thus resulting in an inhibition of the enzyme [35]. Gugle et al. reported the efficacy of topical minoxidil 5% and a combination of topical minoxidil 5%, topical azelaic acid 1.5%, and topical tretinoin 0.01% in the treatment of androgenetic alopecia. They concluded that in both groups, there is a statistically significant increase in hair number and thickness after treatment, with both therapies being equally effective in the treatment of androgenetic alopecia [43]. FGFR2b activity is also induced by androgens, so we think that inhibition of 5-alpha-reductase results in less activity of FGFR2b, with the effects exposed before.

There exists a report where AzA can inhibit plasminogen activator activity [44], but there is no sufficient information that supports this mechanism of action.

Finally, to provide a practical overview of the therapeutic relevance of azelaic acid, we have integrated Table 1, which summarizes its principal mechanisms of action alongside the most common dermatological conditions in which it is clinically applied. This classification is based on the underlying pathophysiological pathways involved, allowing for a clearer understanding of AzA’s multifaceted utility in daily dermatological practice [45].

**Table 1:** Mechanism-based clinical applications of azelaic acid in dermatology

Mechanism of action	Associated dermatological conditions
Anti-inflammatory	Rosacea, perioral dermatitis, hidradenitis suppurativa, psoriasis (as adjunctive therapy)
Antibacterial	Acne vulgaris, folliculitis, perioral dermatitis, hidradenitis suppurativa
Anti-melanogenesis	Melasma, post-inflammatory hyperpigmentation
Antikeratinizing	Acne vulgaris, keratosis pilaris, psoriasis
Free-radical scavenging	Rosacea, psoriasis, post-inflammatory hyperpigmentation, *Alopecia areata* (adjunctive potential)
5-alpha-reductase inhibition	Androgenetic alopecia, acne vulgaris (sebaceous component)

## Conclusions

AzA demonstrates a wide range of therapeutic potential in dermatology, particularly due to its anti-inflammatory, antibacterial, and anti-keratinizing properties. However, while its pharmacological benefits are well documented, further research is needed to fully elucidate its mechanisms of action and optimize its clinical applications in various dermatological disorders.
